# Dataset of exponential growth rate values corresponding non-spherical bubble oscillations under dual-frequency acoustic irradiation

**DOI:** 10.1016/j.dib.2022.107810

**Published:** 2022-01-07

**Authors:** Kálmán Klapcsik

**Affiliations:** Budapest University of Technology and Economics, Faculty of Mechanical Engineering, Department of Hydrodynamic Systems, P.O. Box 91, Budapest 1521, Hungary

**Keywords:** Bubble dynamics, Sonochemistry, Spherical stability, Growth rate, GPU Computing

## Abstract

The dataset described in this paper is related to the paper [1], in which the exponential growth rate values of a spherically oscillating gas bubble modelled by the Keller–Miksis equation are recorded. As the bubble is excited by dual-frequency, the employed parameters are the pressure amplitudes and frequencies corresponding to the first and second harmonic components, respectively, the phase shift between harmonic components, and the equilibrium bubble radius. At each parameter combinations the exponential growth rate values corresponding to mode 2 up to mode 6, and the maximum bubble radius are stored. The huge amount of numerical data are generated that are stored in a public repository [2]. The present paper describes the generated data, the applied numerical model and the implementation details of the program code used to generate the data on graphics processing units (GPUs).

**Specifications Table**

**Table:** 

Subject	Hydrodynamics
Specific subject area	Bubble dynamics and sonochemistry.
Type of data	Figure
	Text files
How data were acquired	Numerical simulations were carried out on GE-Force GTX Titan Black GPUs, by using initial value problem solver algorithm; namely, the Runge–Kutta–Cash–Karp method. For numerical calculations, the MPGOS program package [3], [4] was used.
Data format	Raw: text (txt) files containing numerical results
	Visualized: animation gif, png
Parameters for data collection	The main control parameters are related to the dual-frequency driving; namely, the pressure amplitudes ($P_{A,1}$, $P_{A,2}$) and the frequencies ($f_{1}$, $f_{2}$). Secondary parameters are the equilibrium bubble radius $R_{E}$, and the phase shift θ between the harmonic components. The ambient pressure and temperature were constants $P_{\infty }=1,\mathrm{bar}$ and $T_{\infty }=20\;{}^{\circ }C$, respectively.
Description of data collection	Numerical calculations were carried out on the parameter space of excitation frequencies with a resolution of $f_{1}\times f_{2}=101\times 101$, and the program code loops through all the pressure amplitude combinations, except the unexcited case ($P_{A,1}=P_{A,2}=0$). The total number of investigated parameter combinations is (21×21−1)×(101×101)=4488440 in case of a given bubble size and phase shift. The computations were repeated for different equilibrium bubble sizes between $R_{E}=1-10\;\mu m$ with an increment of 0.5μm. For $R_{E}=3\;\mu m$, the effect of phase shift was also investigated by varying it between 0−1.75π with an increment of 0.25π.
Data source location	Institution:Budapest University of Technology and Economics, Faculty of Mechanical Engineering, Department of Hydrodynamic Systems
	City/Town/Region: Budapest
	Country: Hungary
Data accessibility	Repository name: Mendeley Data
	Data identification number: 10.17632/69jf5ncdmw.1
	Direct URL to data: https://data.mendeley.com/datasets/69jf5ncdmw/1
Related research article	K. Klapcsik, GPU accelerated numerical investigation of the spherical stability of an acoustic cavitation bubble excited by dual-frequency, Ultrason. Sonochem. 77 (2021) 105684.
	doi: https://doi.org/10.1016/j.ultsonch.2021.105684

## Value of the Data

• The shape-stability maps can be used to generate the so-called *bubble habitat* diagrams [5], where the spherical, positional and diffusional stability requirements are fulfilled. The shape stability results are available as part of the present data.
• The results can be used to estimate the chemical yield by using the mathematical formula published in paper [6].
• One can define various quantities such as estimated chemical output per driving intensity that can be a measure of efficiency. Note that the driving intensity is proportional to $P_{A,1}^{2}+P_{A,2}^{2}$. In this way, researchers may be able to enhance efficiency or maximize chemical yield.
• Theoretical researchers in the field of sonochemistry can use the data to elaborate optimization strategies. Experimental researchers can use the data for comparison with measurement.
• Researchers are able to construct mathematical formulas, which describe the growth rate as a function of given parameters by fitting curves to the data [7], [8]. Such kind of mathematical formulas may be used to estimate the shape-stability properties of bubbles.
• Researchers can draw conclusions that are not addressed in the main article. For example, the striped topology of stability maps at lower bubble size implies a relationship between the parametric mode instability and the combination and simultaneous resonances of oscillating bubbles [9], which was not examined yet. By means of the provided program code, one can carry out further computations according to his/her research.

## 1 Data Description

The data repository contains raw and visualised data as well. Raw data files are standard *txt* files containing the numerical results. Visualised data are arrays of figures (stability maps, growth rate values, and an animation) that accompany the research article [1]. As a huge amount of separate data files are generated with different parameters, a proper folder structure and file naming convention is required. These conventions of data file naming and their organization into folder structure is discussed below.

### 1.1 Description of the raw data

The raw data files contain the exponential growth rate corresponding to nonspherical shape modes from mode 2 up to 6, and the maximum radius of bubble oscillation. According to the ultrasonic applications the main investigated parameters are the driving frequencies $f_{1}$, $f_{2}$ and the pressure amplitudes $P_{A,1}$, $P_{A,2}$. The frequency values were varied from 20kHz to 2000kHz and a logarithmic scale was applied. The investigated range was divided into 101 values. The pressure amplitude values were varied between 0 and 2bar with an increment of 0.1bar. The number of pressure amplitude combinations is 21×21−1=440. Note that the non-excited case $P_{A,1}=P_{A,2}=0$ is excluded from the dataset. The raw numerical results for every pressure amplitude combination are saved as text files named as *SphericalStability_PA1_x_PA2_y.txt*, where *x* and *y* denote the value of the pressure amplitude corresponding to the first or second driving components of dual-frequency with two decimal digits precision, respectively.

Each file contains 10,201 rows of numeric data as the resolution of the frequency parameter plane was $f_{1}\times f_{2}=101\times 101=10201$. The columns order are the following. The first six columns are the simulation parameter values; namely, the first signal amplitude $P_{A,1}$, the corresponding frequency $f_{1}$, the second signal amplitude $P_{A,2}$, the corresponding frequency $f_{2}$, the phase shift between θ harmonic component in radians and the equilibrium radius $R_{E}$ in microns. Note that the units of pressure amplitude and frequency values are bar and kHz, respectively. The next two columns contain the dimensionless time values corresponding to the end of transient iterations $\tau_{t}$, and to the end of total simulation $\tau_{T}$, respectively. These two numbers determine the total dimensionless time of averaging. The 9th and 10th columns contain the dimensionless bubble radius and the corresponding dimensionless bubble wall velocity at the end of transient iterations. The columns from 11th up to 15th store the calculated growth rate values corresponding to mode number 2 up to 6. In the last columns, the absolute maximum value of bubble size observed during the investigation of shape stability is recorded. The structure of data files are summarized in Table 1.

**Table 1:** Structure of data files.

$P_{A,1}$	$f_{1}$	$P_{A,2}$	$f_{2}$	θ	$R_{E}$	$\tau_{t}$	$\tau_{T}$	$x_{1}\left(\tau_{t}\right)$	$x_{2}\left(\tau_{t}\right)$	$r_{1,n=2}\ldots r_{1,n=6}$	$x_{1,max}$
bar	kHz	bar	kHz	rad	μm	-	-	-	-	-	-

An example of the data file is given in Table 2. It is worth mentioning that this example is a simplified version of the raw data files that contain all the above mentioned data. From Table 2 one can observe that each of the lines of a data file represents a given $f_{1}-f_{2}$ frequency combination.

**Table 2:** A snippet of the data file named as *SphericalStability_PA1_0.00_PA2_0.10.txt* obtained for equilibrium radius $R_{E}=3\;\mu m$ and phase shift Θ=0.

N	$f_{1},\;\mathrm{kHz}$	$f_{2},\;\mathrm{kHz}$	$r_{1,2}$	$r_{1,3}$	$r_{1,4}$
1	2.0000000000e+01,	2.0000000000e+01,	−4.6277215407e+01,	−8.0573289583e+01,	−1.2495415955e+02,
2	2.0942570961e+01,	2.0000000000e+01,	−4.4194397561e+01,	−7.6946896364e+01,	−1.1933009062e+02,
3	2.1929563923e+01,	2.0000000000e+01,	−4.2205322069e+01,	−7.3483712980e+01,	−1.1395962728e+02,
⋮	⋮	⋮	⋮	⋮	⋮
101	2.0000000000e+03,	2.0000000000e+01,	−4.6277167305e−01,	−8.0583159988e−01,	−1.2492857749e+00,
102	2.0000000000e+01,	2.0942570961e+01,	−4.6288882812e+01,	−8.0572496801e+01,	−1.2494712133e+02,
⋮	⋮	⋮	⋮	⋮	⋮
10201	2.0000000000e+03,	2.0000000000e+03,	−7.5257613557e−01,	−1.1751220448e+00,	−1.6029382251e+00,

It must be mentioned that the growth rate values are calculated with respect to the dimensionless time $\tau =t/f_{1}$; therefore, these values depend on the frequency $f_{1}$ corresponding to the first component of the dual-frequency signal. It is highly advisable to eliminate the frequency dependency when the raw data are used. A possible technique is presented in the research article [1] to rescale these growth rate values:

(1) $$r_{0,n}=r_{1,n}\cdot f_{1}/f_{0},$$

where $r_{1,n}$, is the tabulated growth rate value, $f_{0}$ is the linear eigenfrequency of the system [10]:

(2) $$f_{0}=\frac{1}{2\pi }\sqrt{\frac{3\gamma \left(P_{\infty }-p_{V}\right)}{\rho_{L}R_{E}^{2}}-\frac{2\left(3\gamma -1\right)\sigma }{\rho_{L}R_{E}^{3}}}.$$

**Fig. 1:**
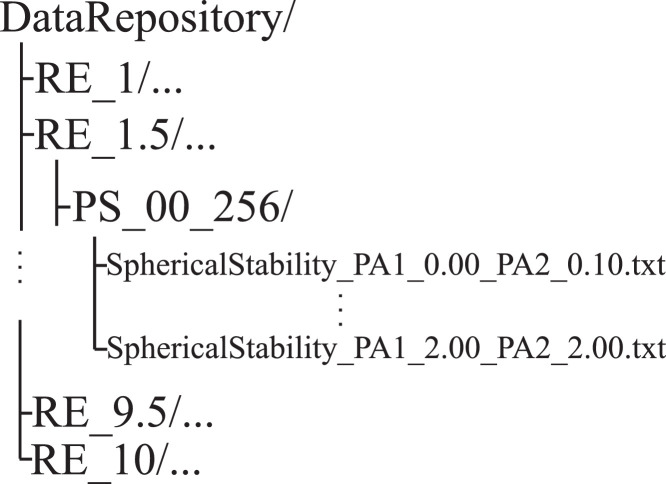
Folder Structure.

The data files obtained in case of a given equilibrium radius and phase shift are organized into the following folder structure*“RE_x/PS_y_256/”. RE* and *PS* stands for equilibrium radius and phase shift, respectively. x denotes the size of the bubble in micron. y means the value of phase shift in radians, eg., “00” means Θ=0πrad or “175” means 1.75πrad. The folder structure is depicted in Fig. 1. The tag “256” in the folder name denotes that the average exponential growth rate was observed over 256 consecutive collapses.

The above described data files are zipped into *“DataRepository.zip”* that is uploaded into the data repository [2].

### 1.2 Description of the visualised data

**Fig. 2:**
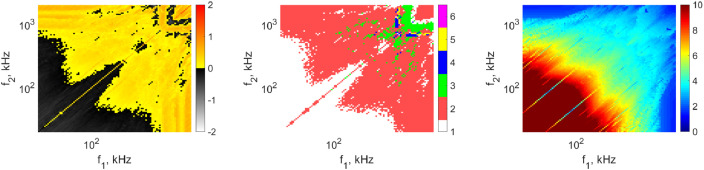
Examples of bi-parametric plots. The left, middle, and right panel show the growth rate corresponding to mode 2, the most unstable mode, and the maximum expansion $R_{\max}/R_{E}$ as the function of frequencies $f_{1}$, $f_{2}$, respectively. Modified from [2].

Numerical data stored in text files named as *SphericalStability_PA1_x_PA2_y.txt* can be visualised as bi-parametric plots as a function of excitation frequencies $f_{1}-f_{2}$. Examples of such bi-parametric plots are given in Fig. 2. Each diagram is obtained at the following parameters: pressure amplitude $P_{A,1}=P_{A,2}=1\;\mathrm{bar}$, equilibrium bubble radius $R_{E}=3\;\mu m$ and phase shift θ=0. The subplots depict the growth rate corresponding to mode number 2, the stability maps and the maximum bubble expansion. Note that the growth rate values are rescaled according to Eq.  (1). In the case of the growth rate, the grey domains denote the shape stable oscillations, and the yellow-red domains denote the shape unstable oscillations. The stability map depicts the most unstable mode at the given pair of frequencies.

Visualised data are zipped into *“FigureRepositor.zip”* file. The zip file contains bi-parametric plots of growth rate, stability maps, and maximum bubble expansion as a function of excitation frequencies $f_{1}-f_{2}$ arranged into arrays of figures.

**Fig. 3:**
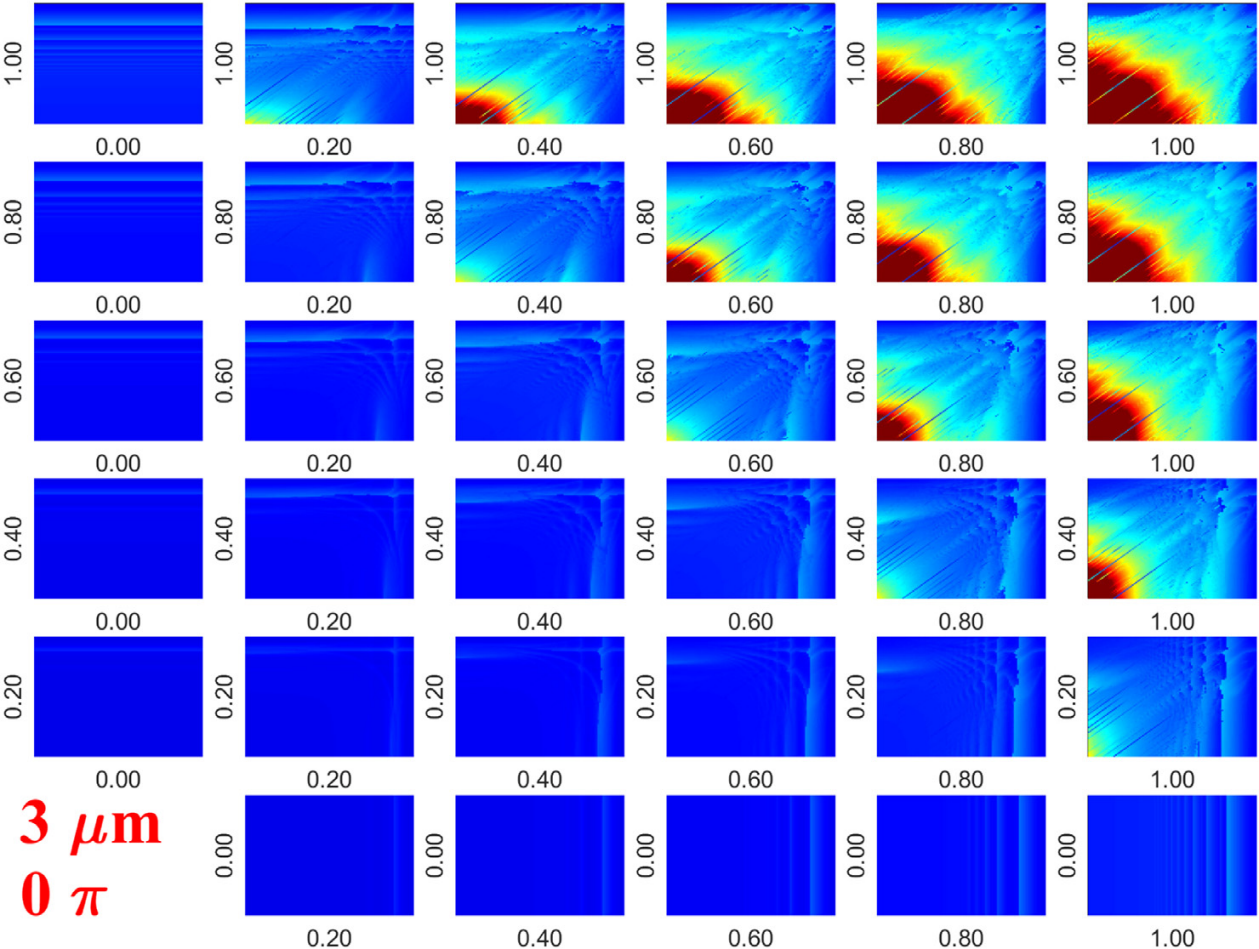
Array of figures of the maximum bubble expansion $R_{\max}/R_{E}$ diagrams. The rows and columns correspond to $P_{A,1}$ and $P_{A,2}$, respectively. Each subplot depicts the maximum bubble expansion as a function of excitation frequencies $f_{1}$ and $f_{2}$ on a logarithmic scale between 20kHz and 2000kHz with a resolution of 101×101.

**Fig. 4:**
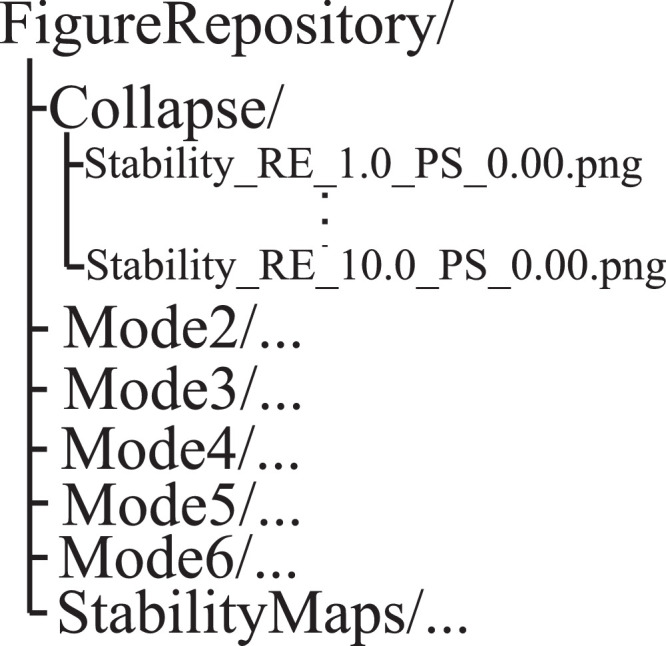
Folder Structure.

For better presentation of the results, such diagrams plotted in Fig. 2 obtained at different pressure amplitude values are arranged into arrays of figures. The columns and rows of arrays correspond to $P_{A,1}$ and $P_{A,2}$ pressure amplitudes, respectively. From left to right, and bottom to up the pressure amplitude values increase with an increment of 0.2rad. An example is given in Fig. 3, where each subplot depicts the maximum bubble expansion $R_{\max}/R_{E}$ as a function of excitation frequencies $f_{1}$ and $f_{2}$. Two more examples of such arrays of figures are plotted in the related research article [1], see Figs.  4 and 5.

These arrays of figures are saved as image files (with png file format), by using the following file naming conventions. The constant parameter values are coded into the file names; therefore, figures are named as *“Stability_RE_x_PS_y.png”*, where *x* is the equilibrium radius with 1 decimal digit precision in micron, and *y* is the phase shift as θ=y·π with two decimal digits precision.

Since the file names are almost identical, the folder structure is important. Folders named as *“Moden/”* contain the figures with average growth rate values corresponding to mode *n, “StabilityMaps/”* contains the stability maps, and *“Collapse/”* contains the figures with maximum bubble radius. The folder structure applied to organize the visual data is depicted in Fig. 4. Note that the colour code in the case of the online figures is the same as in the case of the examples plotted in Fig. 2. These figures are zipped into *“FigureRepository.zip”* that is uploaded into the data repository [2].

### 1.3 Animated gif

Arrays of stability maps obtained at equilibrium bubble radius $R_{E}=3\;\mu m$ for different phase sift θ values between 0−1.75π with an increment of 0.25π were concatenated into an animated gif. The name of the file is *Effect_Of_Theta_Animation_Re_3micron.gif*.

## 2 Experimental Design, Materials and Methods

The spherical stability of a bubble excited by dual-frequency excitation was investigated numerically. The radial dynamics of the bubble was described by the Keller–Miksis bubble model, and the surface wave dynamics was described by a set of independent linear ordinary differential equations. The mathematical model is given in the research paper [1]. The numerical calculations were carried out on GeForce GTX Titan black GPU that has 1707GFLOPS double-precision performance. To exploit the high performance compute capabilities of the GPUs, the MPGOS module called *SingleSystem_PerThread* was used [3], [4]. The module is designed for solving a large number of ordinary differential equation systems in parallel. To solve the system of equations introduced in [1], a separate, dedicated project is created that is available in a public github repository [11]. The repository contains the following files and folders:

• MPGOS/*,
• SphericalStability.cu,
• SphericalStability_SystemDefinition.cuh,
• makefile.

Folder *MPGOS* contains the required header files from the corresponding solver. *SphericalStability.cu* contains the main function that manages the control flow. The kernel execution requires pre-declared device (GPU) functions, such as the model definition (ODEs). The name and definition of these functions can not be modified. These functions are collected in the separate *SphericalStability_SystemDefinition.cuh* header file. The interested readers are referred to the MPGOS manual [3]. The makefile manages the compilation process under a Linux environment.

The code is designed to carry on computation on the parameter plane of excitation frequencies $f_{1}-f_{2}$ and iterating through the pressure amplitude combinations ($P_{A,1}-P_{A,2}$) in case of fixed bubble size and phase shift. For the simulations, from the supported initial value problem solvers, the fourth-order Runge–Kutta–Cash–Karp method with fifth-order estimation (RKCK45) was chosen, which supports adaptive step size control. At the applied version of MPGOS a properly parametrized solver object has to be constructed. These parameters (such as *NT, SD, NCP*... etc.) can be read in the code [11] and their behaviours are described in the MPGOS manual [3]. To reproduce the above described data the parameter values should not be modified. The constructed MPGOS solver object is called *CheckSphericalStability*.

The numerical procedure follows the methodology described in the research article [1]. In what follows, the implementation of this methodology is presented via a simplified snippet of the program code. The outher loop iterates through the pressure amplitude combinations, where *NumberOfSimulationLaunches*=$P_{A,1}\times P_{A,2}=441$. *LaunchCounter = 0* corresponds to the unexcited case ($P_{A,1}=P_{A,2}=0$); thus, it is omitted and the *LauncCounter* starts from 1. *FillSolverObject* function is responsible to define the actual equation parameters, and set the actual state variables and the time domain. The material properties, the equilibrium radius and the phase shift can also be set in this function. Then, these variables are loaded to the GPU memory by means of the member function *SynchroniseFromHostToDevice(All)*. The first for loop inside the main loop handles the transient iterations. Within this loop, the member function *Solve* starts the execution of the GPU kernel that is followed by synchronisations between the CPU (host) and GPU (device). The iterations take place between two consecutive local maximum values of bubble radius, see the research article [1], which is handled by an event implemented in the header *SphericalStability_SystemDefinition.cuh*. In this way, the transient phase is considered as 1024 collapses of a bubble. After the transient iterations, $\tau_{t}$, $x_{1}\left(\tau_{t}\right)$ and $x_{2}\left(\tau_{t}\right)$ variables are collected. The function *PerturbateSolverObject* is responsible to set the initial condition of the surface wave amplitudes $\alpha_{1,n}$ to a fixed value; after that, the integrations run an additional 256 bubble collapses. After the iterations, it is required to copy data back to the host memory from the device memory. As during the iteration, the logarithmic growth corresponding to the surface modes was calculated as

(3) $$R_{n}=\ln\left|\frac{\alpha_{n}\left(\tau^{*}\right)}{\alpha_{n}\left(0\right)}\right|,$$

which is divided by $\tau^{*}=\tau_{T}-\tau_{t}$ to obtain the growth rate values $r_{1,n}=R_{n}/\tau^{*}$. The only remaining task is to write the generated data into the file names as *SphericalStability_PA1_x_PA2_y.txt* according to the present pressure amplitude values.



### 2.1 Customization of the problem

One can modify different parameters in the code according to his/her research. First of all, the resolution of the parameter space can be modified above the *main()* function.



In addition, one can define the lower and upper limit of the parameter domain, and change the applied scale (logarithmic or linear).



The additional constant parameters such as equilibrium bubble radius or the phase shift between the harmonic components can be set within the *FillSolverObject* function. Although the ambient pressure and the polytrophic exponent were constant in the present computations, their values can also be modified.



The material properties are also defined in this function, where *Pv, Rho, ST, Vis* and *CL* stands for vapour pressure $p_{V}$, liquid density $\rho_{L}$, surface tension σ, liquid dynamic viscosity $\mu_{L}$ and sound speed $c_{L}$.

## Ethics Statement

Not relevant.

## CRediT authorship contribution statement

**Kálmán Klapcsik:** Conceptualization, Data curation, Formal analysis, Funding acquisition, Project administration, Investigation, Methodology, Software, Supervision, Validation, Visualization, Writing – original draft.

## Declaration of Competing Interest

The author declares that he has no known competing financial interests or personal relationships that could have appeared to influence the work reported in this paper.
